# The effect of oral immunotherapy treatment in severe IgE mediated milk, peanut, and egg allergy in adults

**DOI:** 10.1002/iid3.218

**Published:** 2018-03-15

**Authors:** Jarkko Mäntylä, Tuuli Thomander, Auli Hakulinen, Kaarina Kukkonen, Kati Palosuo, Helena Voutilainen, Anna Pelkonen, Paula Kauppi

**Affiliations:** ^1^ Respiratory Diseases and Allergology University of Helsinki and Helsinki University Hospital, Skin and Allergy Hospital Helsinki Finland; ^2^ University of Eastern Finland Kuopio Finland

**Keywords:** Desensitization, food allergy, tolerance

## Abstract

**Introduction:**

The standard care of severe food allergy in both adults and children means avoidance of allergens. In recent years promising results of oral immunotherapy (OIT) have been reported in children. In adults, information on OIT in severe food allergy is very limited.

**Objective:**

We aimed to study if OIT is possible in adults.

**Methods:**

We report OIT results in 10 adult patients with milk OIT, nine adult patients with peanut OIT, and four adult patients with egg OIT. The allergy was confirmed with allergen specific IgE tests and oral food challenges (open in milk allergy and double‐blind in peanut and egg allergy). The OIT was performed as open.

**Results:**

The median dose of protein that led to discontinuation of allergen challenge because of symptoms was 7.5 mg in milk allergy, 25 mg in peanut allergy, and 15 mg in egg allergy. The median period of OIT was 515 days. Currently on OIT are 6/10 milk allergic patients, 4/9 peanut allergic patients and 3/4 egg allergic patients. The median dose of milk protein increased by 60‐fold during OIT compared to the allergen challenge dose. In peanut OIT the median dose increased by eightfold and in egg allergy the dose increased with OIT by 35‐fold. Local itching was the most common side effect of OIT (73.9% of the patients), four patients reported having used epinephrine autoinjector and three patients having needed emergency room treatment.

**Conclusions and Clinical Relevance:**

OIT can be given in adult patients with severe milk, peanut, or egg allergy only in selected cases. OIT leads into desensitization but it is not clear whether persistent tolerance can be achieved. Mild adverse events during OIT are common.

## Introduction

The standard care of severe food allergy consists of strict food allergen avoidance and emergency self‐administered drugs (epinephrine injection, oral antihistamines, and glucocorticoid tablets) in case of accidental exposure 1. OIT has been studied as a treatment option with promising results both in milk, peanut and egg allergy in children and adolescents 2, 3, 4, 5, 6. In adults, the information on OIT is mostly lacking.

### Study population

**Figure 1:**
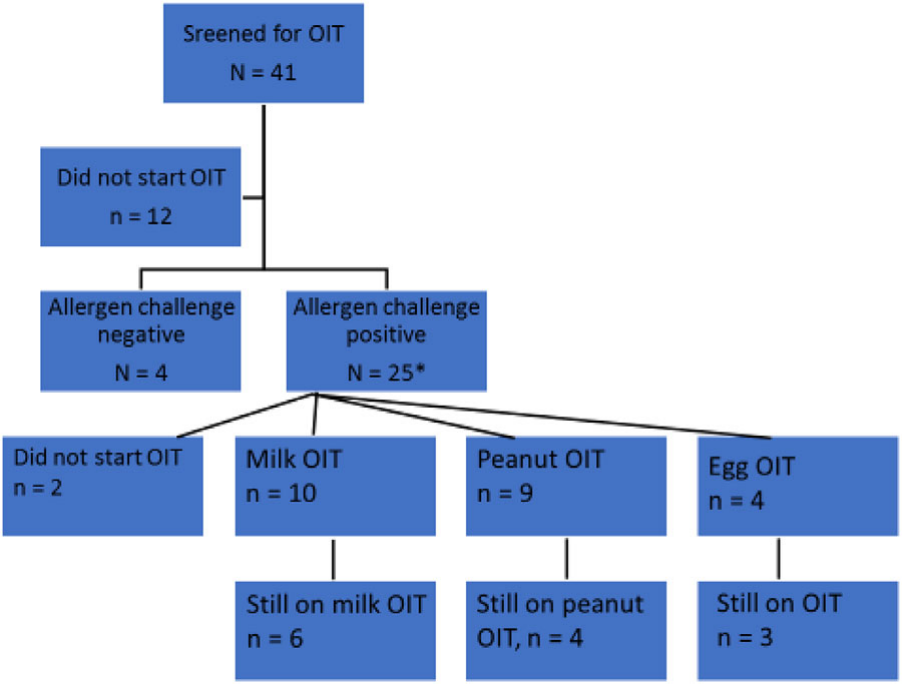
All the patients that were recruited for OIT.

Alltogether 41 adult patients aged 18–64 years have been screened for possible OIT because of severe food allergy (Fig. 1). The diagnosis of food allergy was based on positive history, milk, peanut or egg allergen specific IgE antibodies and oral food challenge test which was open label in milk allergy. All the patients had a clinical history of systemic allergic reaction, emergency medication use and emergency visits because of this food allergy. Of these, 10 patients have started milk OIT, nine patients peanut OIT, and four egg OIT. The study was accepted by the Ethical Committee of Helsinki University Hospital (218/13/03/01/2012) and registered as a clinical trial number HUS21813030112. The patients gave a written informed consent for the study.

Patient history data were collected by questionnaires. All patients underwent a spirometry with a bronchodilatator test, exhaled nitric oxide and a methacholine challenge before 7. Serum samples were taken for specific IgE and allergen component evaluation (Thermo Fisher Scientific, Uppsala, Sweden) before and after OIT.

### Allergen specific challenge tests

For milk allergy, increasing doses were given every 60 min in an open food challenge. The patients were followed for allergic symptoms and treated when necessary with antihistamines (cetirizine 10 mg), prednisolone (40 mg) and epinephrine (0.5 mg im). In peanut and egg allergy, the allergen challenge was performed in a double‐blind manner with increasing doses 6, 8. Stopping of the challenge tests was done early according to the symptoms. All the patients had a clinical history of anaphylaxis, were sensitized (IgE positive) against storage proteins of the food and had a high risk of anaphylaxis in the challenge test. Lack of intensive care unit at our hospital building guided our challenge test performance 9.

### Oral immunotherapy

The OIT was started at the hospital and was continued daily at home. The milk immunotherapy was started with diluted milk (1:10). After the starting dose the dose was increased according to the protocol 10, 11, 12. For peanut OIT, the peanut protein containing margarine was used in the beginning (roasted defatted peanut flour [Byrd Mill, Ashland, VA] was mixed with Keiju margarine [Raisio, Raisio, Finland]) 9. In egg allergy, OIT was started with liquid egg white protein with concentrations of 1 mg/ml and 10 mg/ml and then continued with egg white protein powder (Dava Food Finland, Piispanristi, Finland) 12. During the escalation phase, six of the increased doses in milk OIT, eight of the peanut OIT, and eight of the egg OIT were given at the hospital.

### Statistical analyses

Comparison of IgE tests and age was performed with Mann‐Whitney *U*‐test. Comparison of categoric variables was performed by χ^2^‐test. Comparison of IgE and lung function parameter results during OIT were performed with Wilcoxon signed rank test. The tests were performed with software IBM SPSS Statistics 24 (IBM, Armonk, NY).

## The Results

All the patients with intention to treat with OIT were on average 31 years old and 81% of them were women (Table 1). All the peanut allergic patients reported local itching and irritation in throat and mouth. Egg allergic patients complained stomach pain and nausea more often (28.6% of the patients) than milk allergic (18.2%) or peanut allergic patients (22.2%). The median dose of milk protein in OIT is 450 mg and increased by 60‐fold during OIT. In peanut OIT the median dose is 200 mg of peanut protein and increased by eightfold during OIT. In egg allergy, the median dose in OIT was 525 mg and increased with OIT by 35‐fold. Altogether, the median period on OIT was 515 days.

**Table 1:** Demographics of those who were screened or started OIT and allergen specific IgE values before OIT

	Challenge negative patients	All the screened patients	All who started OIT	Those who quitted OIT	OIT	Challenge negative vs. OIT (p)	Quitted vs. OIT (p)
Age							
Mean (years)	43.0	30.7	31.6	29.3	33.4		
Median	38.0	26.0	33.0	29.5	33.0	0.262	0.740
SD	14.3	11.7	11.3	8.8	13.0		
Gender							
Men	1 (25.0)	8 (19.5)	4 (17.4)	1 (10.0)	3 (23.1)	1.000	0.315
Women	3 (75.0)	33 (80.5)	19 (82.6)	9 (90.0)	10 (76.9)		
S‐IgE milk (kU/L)							
Mean	1.3	86.2	97.6	72.9	117.4		
Median	1.6	53.3	54.7	53.3	67.4	0.057	1.000
SD	0.9	105.2	113.5	74.2	143.4		
S‐IgE peanut (kU/L)							
Mean		88.9	116.2	11.6	220.9		
Median		23.5	19.9	8.8	54.6		0.057
SD		213.0	261.5	10.7	360.9		
S‐IgE egg white (kU/L)							
Mean	4.0	45.7	81.3	6.8	106.1		
Median	4.0	14.5	78.2	6.8	140.0	0.500	0.500
SD		65.4	81.1		78.5		
S‐IgE (kU/L)							
Mean	877.0	1162.6	1455.7	1385.0	1508.8		
Median	295.0	433.0	343.5	121.0	1205.5	0.630	0.059
SD	1209.1	1744.3	2235.7	3132.9	1504.9		
Allergic rhinitis							
*n* (%)	3 (75.0)	28 (68.3)	19 (82.6)	7 (70.0)	12 (92.3)	0.383	0.231
Atopic eczema							
*n* (%)	3 (75.0)	24 (58.5)	16 (69.6)	5 (55.6)	11 (84.6)	0.712	0.221
Asthma							
*n* (%)	3 (75.0)	26 (63.4)	16 (69.6)	6 (60.0)	10 (76.9)	1.000	0.554

The patients were allowed to continue the OIT after one year and thus of 23 patients with OIT, 13 patients (56.5%) are still on OIT (six patients (60%) of milk OIT patients, four patients (44%) of peanut OIT, and three patients (75%) on egg white OIT are currently on OIT (Table 1). Milk allergic patients had a median of 54.7 kU/L allergen specific IgE antibodies before OIT, 30.3 kU/L one year after OIT and 10.7 kU/L in 2017 (Table 2). Peanut allergic patients had a median of 19.9 kU/L allergen specific IgE antibodies before OIT, and 32.0 kU/L one year after OIT (Table 2). However, the average allergen specific IgE in the peanut allergic group decreased from 116.2 kU/L, into 32.4 kU/L at one year. Likewise, IgE antibodies against *Ara h 2* decreased from an average of 48.8 to 33.0 kU/L at one year and 36.4 kU/L in 2017. Egg allergic patients had a median of 78.2 kU/L allergen specific IgE antibodies before OIT, 46.1 kU/L one year after OIT (Table 2).

**Table 2:** Allergen specific IgE values in milk allergic patients with milk OIT before OIT and at one year of OIT

	Before OIT	At 1 year	2017	Before vs 1 year (p)
Milk allergy and milk OIT				
S‐IgE milk (kU/L)				
Mean	97.6	29.3	11.0	
Median	54.7	30.3	10.7	0.225
SD	113.5	18.3	8.6	
S‐IgE casein (kU/L)				
Mean	57.8	19.5	12.4	
Median	41	14.4	10.6	0.068
SD	60.4	17.9	11.2	
S‐IgE lactalb (kU/L)				
Mean	6.2	3.6	1.7	
Median	3.1	3.0	1.6	0.109
SD	7.3	4.2	1.9	
S‐IgE β‐lactogl (kU/L)				
Mean	5.8	2.1	0.7	
Median	2.0	0.4	0.3	0.285
SD	9.5	3.1	1.0	
S‐IgE (kU/L)				
Mean	2201.9	1177.3	899.0	
Median	464.0	1051.0	899.0	0.18
SD	2953.5	653.7	534.6	

Peanut allergy and peanut OIT				
S‐IgE peanut (kU/L)				
Mean	116.2	32.4	59.2	
Median	19.9	32	50.9	0.285
SD	261.5	27.4	36.8	
S‐IgE rArah2 (kU/L)				
Mean	48.8	33.0	36.4	
Median	10.6	26.8	24.0	0.893
SD	104.1	34.5	22.7	
S‐IgE rArah1 (kU/L)				
Mean	9.1	14.7	13.8	
Median	3.0	7.4	8.4	0.273
SD	19.7	22.7	11.7	
S‐IgE rArah3 (kU/L)				
Mean	7.8	8.4	9.6	
Median	0.4	0.5	4.1	0.593
SD	19.9	16.1	12.8	
S‐IgE rArah9 (kU/L)				
Mean	0.5	0.4	0.7	
Median	0.0	0.1	0.5	0.109
SD	1.2	0.8	0.7	
S‐IgE rArah8 (kU/L)				
Mean	2.7	1.4	0.1	
Median	0.0	0.1	0.1	1
SD	3.5	1.9	0.1	
S‐IgE (kU/L)				
Mean	522.8	549.5	975.0	
Median	95.5	549.5	975.0	
SD	892.4	613.1		

Egg white allergy and egg OIT				
S‐IgE egg white (kU/L)				
Mean	81.3	47.3	32.8	
Median	78.2	46.1	32.8	0.285
SD	81.1	40.7		
S‐IgE ovomucoid (kU/L)				
Mean	22.4	28.1	12.9	
Median	20.2	20.7	12.9	0.593
SD	15.5	22.9		
S‐IgE conalbumin (kU/L)				
Mean	46.5	30.6		
Median	46.5	17.0		0.317
SD		39.1		
S‐IgE ovalbumin (kU/L)				
Mean	38.5	20.1	15.7	
Median	38.0	24.5	15.7	0.285
SD	38.0	13.7		
S‐IgE (kU/L)				
Mean	958.7	5624.5		
Median	551.0	5624.5		0.317
SD	1032.7	7563.9		

The respective results for specific IgE values for peanut OIT and egg white OIT.

The OIT did not affect statistically significantly on the lung function parameters (data not shown).

### Adverse effects

Oral and throat itching was the most common side effect of OIT (73.9% of the patients). Four patients (17.4%) reported having used epinephrine autoinjector and three patients (13%) having needed emergency room treatment of all the 23 patients having immunotherapy for either milk, peanut or egg allergy. Epinephrine autoinjector was used at least once by three patients (30.0%) with milk OIT and by one patient (11.1%) with peanut OIT. Four of the milk OIT patients discontinued because of allergic symptoms during escalation phase. One had an emergency visit caused by generalized urticaria. One peanut OIT patient discontinued because of pregnancy during escalation phase. Four peanut OIT patients discontinued because of allergic symptoms during escalation phase. One had flush skin symptoms and stomach pain when changing from peanut margarine (dose 50 mg peanut protein) into peanuts (1/4 peanut) at the hospital. Adverse effects led to discontinuation in one egg OIT patient (exacerbation of atopic dermatitis).

## Discussion

We report altogether 23 adult patients having started OIT for severe milk, peanut or egg allergy and of these 13 patients are still on immunotherapy. Median length of OIT has been 515 days (SD 754 days). Allergen specific IgE values have decreased in all the food groups during OIT. Mild allergic reactions in all the food OIT groups have been common. 17.4% of all the OIT patients had used epinephrine autoinjector but all the reactions have been managed successfully.

In a Cochrane review for milk OIT, all the study individuals were children contrary to our adult population. In addition, different OIT protocols were reported making comparison of the studies difficult 13. In the recent systematic review and meta‐analysis of allergen immunotherapy for food allergy, altogether 1259 patients were analysed (mostly children) 14. OIT was effective and increased the threshold for symptoms but was associated with a modest increased risk of serious systemic adverse reactions. Further, the new EAACI guidelines on immunotherapy advises to perform OIT in research centers or in clinical centers with extensive experience in OIT and that the patients should be provided with information about OIT to be able to do an informed decision about the therapy 15.

Strengths of the study is the use of oral immunotherapy with food products instead of epicutaneous or sublingual products both of which are known to be less effective than OIT. In addition, the real life setting shows that some individual patients with severe food allergy are willing to seek for treatment that would decrease risk of severe allergic reactions in every‐day life. We had both milk, peanut and egg white allergic patients and OIT in the study. Although the study population is small, we have relatively long OIT length especially in the milk allergic group (median 672 days). Weakness of the study is the small study population although in phase 2 trials as this the study population is always small. Further, lack of control group makes it more difficult to estimate the study results.

## Conclusions

OIT can be given in adult patients with milk, peanut or egg allergy in individual cases. This therapeutic option is not ready to be included in the everyday clinical practice and thus we propose OIT in adults to be performed in clinical trials only. Further, according to these preliminary results, achieving persistent tolerance and cure in adults seems unlikely but alleviating allergy may be achieved.

## Clinical Implications

OIT can be given in adult patients with severe milk, peanut or egg allergy only in selected cases. OIT leads into desensitization but it is not clear whether persistent tolerance can be achieved. Mild adverse events during OIT are common.
